# APOBEC3B is overexpressed in cervical cancer and promotes the proliferation of cervical cancer cells through apoptosis, cell cycle, and p53 pathway

**DOI:** 10.3389/fonc.2022.864889

**Published:** 2022-09-29

**Authors:** Zhi Wei, Jianfeng Gan, Xuan Feng, Mo Zhang, Zhixian Chen, Hongbo Zhao, Yan Du

**Affiliations:** ^1^ Shanghai Key Laboratory of Female Reproductive Endocrine Related Diseases, Obstetrics and Gynecology Hospital, Fudan University, Shanghai, China; ^2^ Department of Obstetrics and Gynecology of Shanghai Medical School, Fudan University, Shanghai, China; ^3^ Institutes of Integrative Medicine, Fudan University, Shanghai, China

**Keywords:** APOBEC3B expression, cervical cancer, proliferation, apoptosis, chemoresistance

## Abstract

**Objective:**

*APOBEC3B* (*A3B*), a member of the APOBEC family of cytidine deaminases, has been gradually regarded as a key cancerous regulator. However, its expression and mechanism in cervical cancer (CC) have not been fully elucidated. This study was to investigate its expression pattern and potential mechanism on the cell cycle, as well as HPV oncogenes in CC.

**Methods:**

Data from The Cancer Genome Atlas (TCGA) and Gene Expression (GEO) were used to indicate the mRNA expression pattern of A3B in cervical cancer. Western blot assay was used to detect A3B levels in SiHa and Hela cell lines. Immunohistochemistry (IHC) was used to explore A3B protein abundance and sublocation in cervical cancer as well as normal cervical tissues. Based on the Protein atlas (www.proteinatlas.org), A3B expression in the SiHa cell line is lower than in the HeLa cell line. Therefore, the SiHa cell line was used for *A3B* gene overexpression experiments while the HeLa cell line was used for knockdown experiments. Flow cytometry analysis was used to detect cell apoptosis. Biological function and cancer-related pathways of *A3B* were conducted using bioinformatics analysis.

**Results:**

*A3B* mRNA was significantly overexpressed in cervical cancer in TCGA-cervical squamous cell carcinoma and endocervical adenocarcinoma (CESC), GSE67522, and GSE7803. A3B was more highly expressed in cervical cancers than in high-grade squamous intraepithelial lesions and normal controls. A3B expression was found to be progressively activated during cervical cancer development. IHC results showed that A3B was significantly higher in cervical cancer tissues than in normal cervical tissues. *A3B* plasmid-mediated overexpression experiments and *A3B* siRNA-mediated knockdown experiments showed that A3B significantly promotes cell proliferation, migration, cell cycle, and chemoresistance in cervical cancer cells by the p53 pathway. GO and KEGG analyses showed that A3B expression was strikingly associated with cell proliferation, apoptosis, and immune-associated pathways.

**Conclusions:**

Taken together, our study implies that A3B promotes cell proliferation, migration, and cell cycle and inhibits cancer cell apoptosis through the p53-mediated signaling pathway. Moreover, A3B could also contribute to chemoresistance in cervical cancer cells. It may be a potential diagnostic biomarker and therapeutic target for chemoresistant cervical cancers.

## Introduction

Cervical cancer is one of the most common gynecologic cancers among women and is a leading cause of female cancer-related deaths worldwide. Global cancer statistics have reported that there were about 570,000 newly diagnosed invasive cervical cancer cases and about 311,000 cases of death caused by cervical cancer in 2018 (1). About 95% of cervical cancer cases are caused by persistent infection with certain carcinogenic human papillomavirus (HPV) genotypes (2). HPV infection remains one of the most prevalent sexually transmitted diseases worldwide, with over 14 million individuals infected annually (3). In addition, prophylactic HPV vaccines have limited effects in eliminating pre-existing infections, thus cervical cancer is and will remain to be an important health burden (4). Therefore, it is important to investigate the molecular mechanisms and key regulators of cervical cancer progression and prognosis in order to identify novel biomarkers for individualized prevention and treatment strategies.

Apolipoprotein B mRNA-editing enzyme catalytic polypeptide 3 members (APOBEC3s) are a family of cytidine deaminases. APOBEC3s are responsible for the majority of cytosine mutations and can specifically edit DNA/RNA through irreversible cytidine and deoxycytidine deamination, resulting in the conversion of target cytosine (C) to uracil (U) and consequently causing DNA/RNA changes/damages (5, 6). The Cancer Genome Atlas (TCGA) project found that among invasive cervical cancer patients, the APOBEC mutation load was strongly correlated with the total number of mutations per sample, indicating that APOBEC mutagenesis is the predominant source of mutations (6). Previous studies have shown that APOBEC3B is associated with malignant transformation (7). It is speculated that APOBEC3B could induce genome instability in various cancers (8). Evidence has shown that APOBEC3B expression is upregulated by inflammatory factors and plays an important role in the genesis and progression of virus-associated cancers, such as hepatocellular carcinoma (5, 9).

It was found that APOBEC3B (A3B) is an important source of genomic mutations in a variety of human cancers, including breast cancer, head and neck cancer, cervical cancer, bladder cancer, lung cancer, and ovarian cancer. In addition, the E6 oncoprotein in high-risk HPV can affect A3B expression (10). Another study discovered significant differences in the expression of EGFR/PI3K/Akt/mTOR signaling-related proteins and clinical prognosis in patients with HPV16-positive or HPV16-negative head and neck squamous cell carcinoma (HNSCC) (11). The overexpression of A3B in HPV-positive tumors is caused by the viral E6/E7 oncoprotein and may be an early switch in the tumorigenic process. A3B expression was found to be progressively activated during HPV-negative tumor development and suggested that A3B overexpression may provide a marker for advanced oral heterogeneous hyperplasia and cancer (12).

Studies have shown that in normal breast epithelial cells stably transfected with HPV18, a significant upregulation of A3B was observed, and related experimental results also revealed that HPV may participate in the early stages of breast cancer *via* A3B (13). Moreover, A3B expression has been found to be higher in breast cancer tissues than in noncancerous tissues associated with lymph node metastasis and nuclear grading and is a reliable phenotypic marker of invasiveness in breast cancer (14). The study has reported that A3B is elevated in cervical cancer cells (15). Moreover, high-risk HPVE6 and HPVE7 proteins, which bind to and degrade p53 and pRB, are essential proteins in the molecular pathogenesis of cervical cancers (16). Research has found that the E6 protein of p53-bound oncogenic HPV stimulates the degradation of p53. The E6 protein of HPV targeted p53 and led to its degeneration, thereby promoting carcinogenesis (17, 18).

The current study aimed to investigate the expression pattern of APOBEC3B (A3B) in cervical cancers and the possible molecular mechanisms of APOBEC3B on the cell cycle as well as HPV oncogenes in cervical cancer.

## Materials and methods

### Bioinformatics analysis

The Tumor Immune Estimation Resource (TIMER, version 2.0) (http://timer.cistrome.org/) “exploration” module was used to display the differential gene expression of APOBEC3B in various tumor tissues and corresponding normal tissues (19).

Tumoral RNA-seq data of cervical cancer patients as well as mRNA expression data of paired normal tissue samples were from TCGA database and were downloaded from the Genomic Data Commons (GDC) data portal. Other data of normal tissue samples were obtained from the normal cervix in the GTEx V8 release version (https://gtexportal.org/home/datasets). A complete description of information for each sample was described in the GTEx official annotation (20). The GSE67522 and GSE7803 datasets used are from the GEO database (https://www.ncbi.nlm.nih.gov/geo/), and the downloaded data format is MINIML. Analyses were performed after grouping the clinical phenotype. The box plot was implemented by the R software package ggplot2, and heatmap was displayed by the R software package pheatmap.

### Differential genes and functional analysis

We used the Limma package (version: 3.40.2) of R software to study the differential expression of mRNAs in the cervical cancer database. The adjusted *p*-value was calculated to correct data in TCGA. The following criteria were used to screen for differential genes in TCGA dataset: “Adjusted *p* < 0.05 and Log (fold change) >2 or Log (fold change) <−2.” Based on the expression level of APOBEC3B, we divided TCGA samples into A3B^low^ (<25% of the quantile distribution) and A3B^high^ (>75% of the quantile distribution) expression groups. To further confirm the underlying function of potential targets, the data were analyzed by functional enrichment. Gene Ontology (GO) is a tool for annotating genes with functions, especially molecular functions, biological pathways, and cellular components. The Kyoto Encyclopedia of Genes and Genomes (KEGG) pathway enrichment analysis is a practical resource for the analytical study of gene functions and associated high-level genome functional information. To better understand the carcinogenesis of mRNA, the ClusterProfiler package (version: 3.18.0) in R was employed (21). We searched “cervical cancer” in NCBI (https://www.ncbi.nlm.nih.gov/gene). GSE26511 samples were divided into A3B^high^ expression group (*n* = 20) and A3B^low^ expression group (*n* = 19) and were analyzed using the criterion “*p* < 0.05 and Log (fold change) >1.5 or Log (fold change) <−1.5” (21). The common upregulated genes were obtained to perform the functional analysis. KEGG analysis and GO analysis were conducted using the common upregulated genes with Metascape (https://metascape.org/).

For the Cancer Genome Atlas (TCGA) database, we downloaded tumor RNA-seq (FPKM) from the GDC. We used the one-class logistic regression (OCLR) score algorithm constructed by Malta et al. to calculate mRNAsi. We used the Spearman correlation (RNA expression data), and then subtracted the minimum value and divided it by the linear transformation of the maximum value, mapping the dryness index to the range. All the above analyses were performed using the R package by the R Foundation for Statistical Computing (2020), version 4.0.3.

### Immunohistochemistry

Tumor tissues and normal adjacent tissues of five cervical cancer patients were obtained from the Tissue Bank of the Obstetrics and Gynecology Hospital of Fudan University.

Tissue microarrays (TMAs) of cervical cancer specimens were obtained from the Tissue Bank of the Obstetrics and Gynecology Hospital of Fudan University. TMAs were constructed following previous protocols (22, 23). After TMA construction, a hematoxylin and eosin (HE) section of the recipient block was reviewed to confirm that the cores contained the intended region. TMA cutting was performed, and finished slides were embedded in paraffin for preservation at 4°C before immunohistochemistry assays.

The de-paraffinized sections were incubated with 20% goat serum for 30 min to block nonspecific binding and were then incubated with primary antibodies against APOBEC3B (Abcam, Cambridge, UK, 1:100) at 4°C overnight followed by an anti-rabbit secondary antibody (1:100) for 1 h at 37°C. Bound antibody was then visualized using the EnVision™ Detection Systems (Dako, Glostrup, Denmark). The expression of APOBEC3B was examined using immunohistochemistry according to the previous protocol (22, 23). Disagreements were settled by consensus. The study protocol was approved by the institutional review board of the hospital. All patients provided written informed consent.

### Cell culture and cell lines

The human cervical cancer cell lines HeLa and SiHa were obtained from MD Anderson Cancer Center, and the original source is the American Type Culture Collection (ATCC; Manassas, VA, USA). HeLa cells were cultured in 1640 complete medium and DMEM/high-glucose medium supplemented with 10% FBS in 5% CO_2_ at 37°C, respectively. SiHa cells were cultured in DMEM/high-glucose medium in the same condition.

### Transfection of cervical cancer with plasmids and siRNA

Based on the Protein Atlas (www.proteinatlas.org), the SiHa cell line has lower A3B expression relative to the HeLa cell line (**Supplementary Figure S1D**). Two cervical cancer cell lines (HeLa and SiHa) with different endogenous expressions of A3B were chosen. HeLa and SiHa cells were seeded in the 60-mm-diameter dishes at a density of 2 × 10^5^/well. SiHa cells were transfected with vector construct or APOBEC3B overexpression construct, while HeLa cells were transfected with APOBEC3B siRNA or scrambled siRNAs by Lipo2000 reagent according to the manufacturer’s instructions. The siRNA targeting APOBEC3B and scrambled siRNAs were synthesized and purified by RioBio Co. (Guangzhou, China). The APOBEC3B overexpression plasmid and vector constructs were obtained from Asia-Vector Biotechnology (Shanghai, China). Each experiment was repeated three times.

### Western blot analysis

After 48 h of transfection, all transfected HeLa and SiHa cells were collected and lysed in 1× SDS lysis buffer (50 mM of Tris-HCl, pH 6.8, 2% SDS, 10% glycerol, 1 mM of Na_3_VO4, and 1 mM of PMSF); total protein was quantified by the BCA method. A total of 40 μg of protein per lane was loaded on an SDS-PAGE gel and transferred to a PVDF membrane (Millipore Corporation, USA), which was blocked with PBS containing 5% BSA and 0.05% Tween 20. The membrane was incubated with specific primary antibodies and followed by incubation with HRP-conjugated secondary antibodies (Jackson ImmunoResearch Laboratories, West Grove, PA, USA). The labeled proteins were then visualized using fluorography using an enhanced chemiluminescence system (Thermo Scientific, Pierce Biotechnology, USA). Each experiment was repeated three times.

### CCK8 assays

Relative cell viability was assayed by Cell Counting Kit-8 (CK04, Dojindo Laboratories) according to the manufacturer’s recommendation. HeLa and SiHa cells were grown in 96-well plates at a density of 3 × 10^3^/well and then transfected with APOBEC3B construct or siRNAs for the indicated time. In total, 20 µl of CCK-8 reagent was added to the cells and incubated for 1 h. The absorbance at 450 nm was measured by Multiskan Spectrum (Spectra Max190, Molecular Devices). Six replication experiments were performed.

### Cell migration assay

The transwell chamber placed into the 24-well culture plate was called the upper chamber, and the culture plate was called the lower chamber. The cells were incubated in a serum-free medium and were seeded in the upper chamber; a complete medium containing 10% FBS is generally added to the lower chamber. The cells were fixed with 4% paraformaldehyde and then stained with 0.1% crystal violet (Solarbio, China) after 24 h of the migration experiment.

### Flow cytometry analysis

Cells were synchronized by serum starvation for 24 h and cultured in a complete medium for 24 h. Cells were then digested with 0.25% trypsin fixed in 75% ethanol. For PI staining, cells were incubated in the presence of 40 µg/ml PI (Molecular Probes) and 250 µg/ml RNase A (Roche Diagnostics Ltd.) at 37°C for 30 min. Progression into the different cell cycle phases was calculated by propidium iodide staining. The percentage of the positive cells and cell cycle progression in each sample were measured *via* flow cytometry. Each experiment was repeated three times.

### Combined cisplatin drug analysis

In total, 3,000 cells (100 µl, cell suspension density: 3 * 10^4^/ml) were placed into each well of a 96-well plate. PBS solution was added around the 96-well plate to prevent loss of edge medium; 12 h after cell adhesion, the complete medium was replaced with cisplatin concentrations of 5, 10, 20, and 30 μM for 24, 48, and 96 h, respectively. Cell viability was then measured by the CCK8 assay as described above.

### Statistical analysis

Triplicate assays were repeated in all molecular biology experiments. Experimental data are presented as the mean ± SD. The differences between the groups were analyzed *via* Student’s *t*-test and Mann–Whitney *U* test. A two-sided *p* < 0.05 was designated as statistically significant. All analyses were performed using GraphPad (version 8.4.3).

## Results

### Expression of APOBEC3B in different patients using bioinformatics analysis and immunohistochemistry

**Figure 1:**
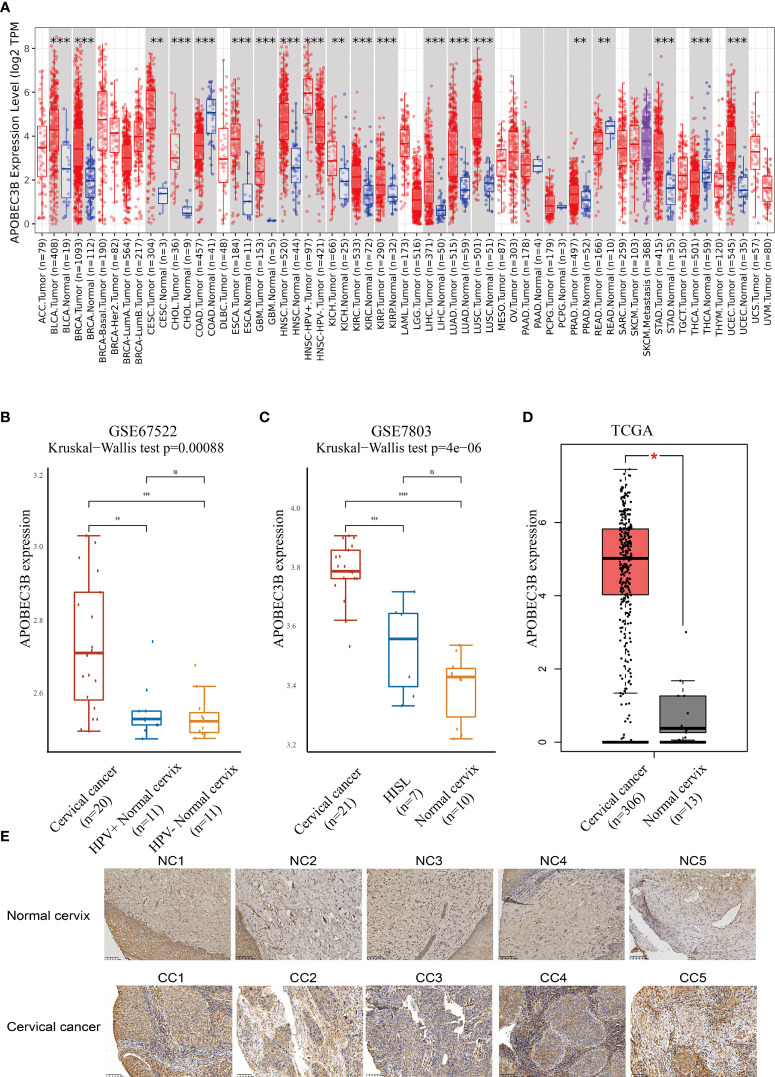
APOBEC3B was highly expressed in tumor tissues. **(A)** Expression levels of A3B in different TCGA cancer types. A3B levels were significantly higher than normal tissue in various cancers in TCGA database. **(B)** Cervical cancer expressed A3B at a higher level compared with HPV+ and HPV− normal cervix in GSE67522. **(C)** Cervical cancer had higher A3B expression compared to HISL and normal cervix in GSE7803. **(D)** A3B showed a higher expression of cervical cancer than the normal cervix. Data are presented as the mean ± SD, and results were analyzed by a two-sided Student’s *t*-test. **(E)** High A3B expression in cervical cancer tissue. ^*^ *p* < 0.05; ^**^ *p* < 0.01; ^***^ *p* < 0.001. ^****^ *p* < 0.0001; ns, no significance.

The expression of APOBEC3B was evaluated in different tumor types and adjacent normal tissues using the TIMER database. As shown in **Figure 1A**, the APOBEC3B expression level was significantly higher compared with adjacent normal tissues in a variety of tumors. In addition, the expression of APOBEC3B was significantly higher in HPV-positive HNSC compared with HPV-negative HNSC. However, in colon adenocarcinoma (COAD), rectum adenocarcinoma (READ), and thyroid carcinoma (THCA), the expression level of APOBEC3B was significantly lower than that in adjacent normal tissues.

We further found that APOBEC3B expression was higher in HPV16-positive cervical cancers than in HPV16-positive nonmalignant tissues and HPV-negative histologically normal controls in GSE67522 (**Figure 1B**). Moreover, APOBEC3B was also highly expressed in cervical cancers than in high-grade squamous intraepithelial lesions and normal controls in GSE7803 (**Figure 1C**). TCGA database showed similar results (**Figure 1D**). In summary, all results showed that APOBEC3B is markedly elevated in cervical cancer tissues. This finding was further verified by the immunohistochemical results of our clinical samples (**Figure 1E**). In cervical cancers, APOBEC3B expression was strongly positive compared with the normal cervix.

### Functional enrichment analysis of A3B in TCGA database

**Figure 2:**
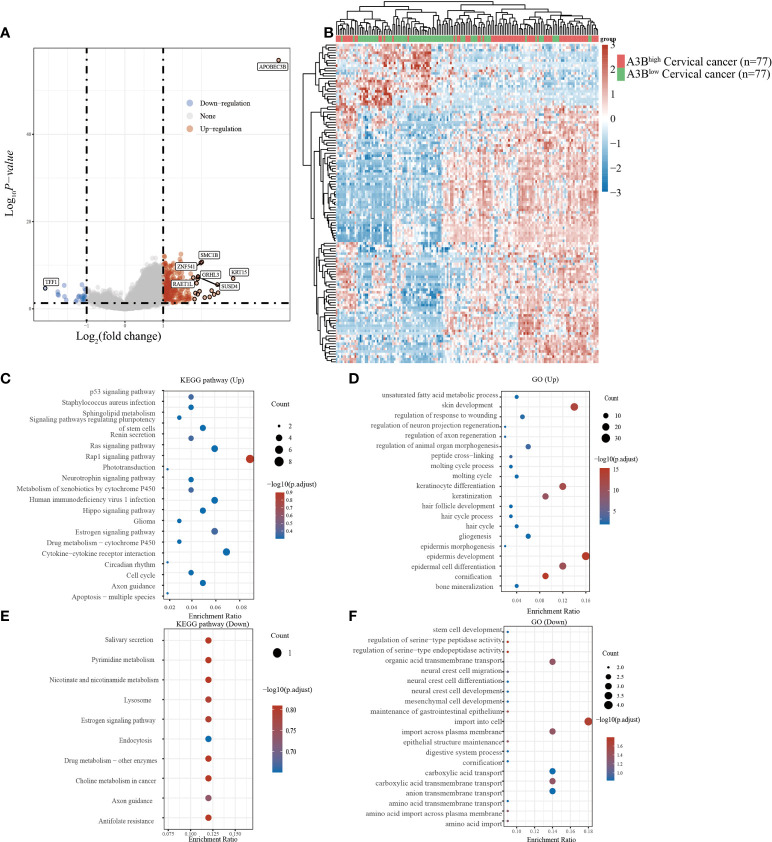
Different gene expression patterns and enrichment analyses of APOBEC3B^high^ and APOBEC3B^low^ in expression cervical cancer in TCGA. **(A)** A volcano plot for differential gene expression in TCGA samples with high and low A3B expression. In total, 124 upregulated genes and 27 downregulated genes were identified. **(B)** The heatmap analysis of differential genes. **(C)** KEGG pathway analysis of upregulated genes. **(D)** GO analysis of upregulated genes. **(E)** KEGG pathway analysis of downregulated genes. **(F)** GO analysis of downregulated genes.

Cervical cancer cases in TCGA were divided into two groups based on the high and low expression of APOBEC3B following previous methods. In total, 236 upregulated genes and 24 downregulated genes (**Figures 2A, B** **)** were identified in TCGA dataset. Studies have found that *TFF1*, one of the downregulated genes in the A3B^high^ group, was regulated by APOBEC3B in breast cancers (24). We then performed GO and KEGG analyses to evaluate the function of those altered genes. GO analysis revealed that the upregulated genes with APOBEC3B were significantly enriched in epidermis development (**Figure 2D**), and the downregulated genes were significantly enriched in cell import (**Figure 2F**). Moreover, KEGG analysis demonstrated that upregulated genes with APOBEC3B were mostly enriched in the Rap1 signaling pathway, estrogen signaling pathways, and cytokine–cytokine receptor interaction (**Figure 2C**). Moreover, downregulated genes for KEGG analysis also showed that estrogen signaling pathways were significantly enriched (**Figure 2E**).

### Functional enrichment analysis of A3B in the GEO database

**Figure 3:**
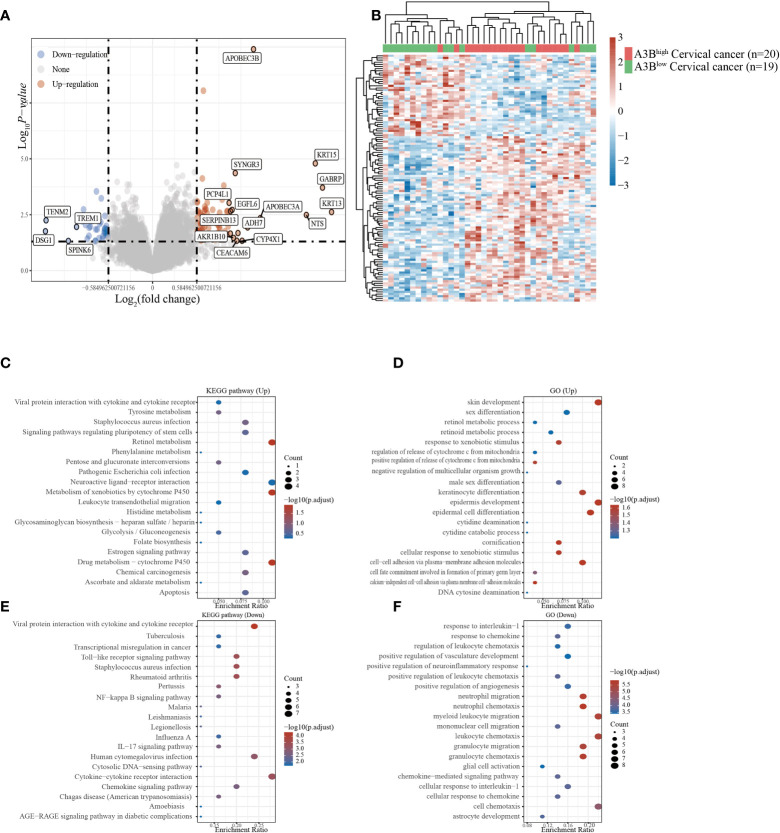
Different gene expression patterns and enrichment analyses of APOBEC3B^high^ and APOBEC3B^low^ expression in cervical cancer in GSE26511. **(A)** A volcano plot for differential gene expression in high and low A3B expression in GSE26511. In total, 78 upregulated genes and 38 downregulated genes were identified. **(B)** The heatmap analysis of differential genes. **(C)** KEGG pathway analysis of upregulated genes. **(D)** Analysis of upregulated genes. **(E)** KEGG pathway analysis of downregulated genes. **(F)** GO analysis of downregulated genes.

Cervical cancer cases in GSE26511 were divided into two groups based on the high and low expression of APOBEC3B as described before. In total, 78 upregulated genes and 38 downregulated genes (**Figures 3A, B** **)** were identified in the GSE26511 dataset. We then performed GO and KEGG analyses to evaluate the function of those altered genes. GO analysis revealed that the genes, upregulated by APOBEC3B were significantly enriched in epidermis development and skin development (**Figure 3D**), and the downregulated genes with APOBEC3B were significantly enriched in myeloid leukocyte migration, leukocyte chemotaxis, as well as cell chemotaxis (**Figure 3F**). Moreover, KEGG analysis demonstrated that upregulated genes with APOBEC3B were mostly enriched in drug metabolism–cytochrome P450 (**Figure 3C**). Moreover, downregulated genes for KEGG analysis also showed that cytokine–cytokine receptor interactions were significantly enriched (**Figure 3E**).

Followed by the Venn diagram analysis, 39 genes were both upregulated from differential expression genes (DEGs) of A3B^low^ and A3B^high^ in TCGA dataset and DEGs of A3B^low^ and A3B^high^ in the GSE26511 dataset (**Supplementary Figure S1B**). Consistently, the gene enrichment analyses showed that upregulated genes were enriched in pathways related to cell fate commitment and regulation of extrinsic apoptotic signaling pathways (**Supplementary Figure S1C**). We further analyzed the relativity between one-class logistic regression (OCLR) and APOBEC3B gene expression, and our results revealed that tumor tissues with APOBEC3B high expression have higher stemness, which evaluates the proliferative activity and malignant potential of tumor cells (**Supplementary Figure S1A**). All suggest that A3B is likely to serve as an important factor in the development of cervical carcinogenesis in cervical cancer.

### APOBEC3B expression could promote cell migration ability and inhibit cell apoptosis

**Figure 4:**
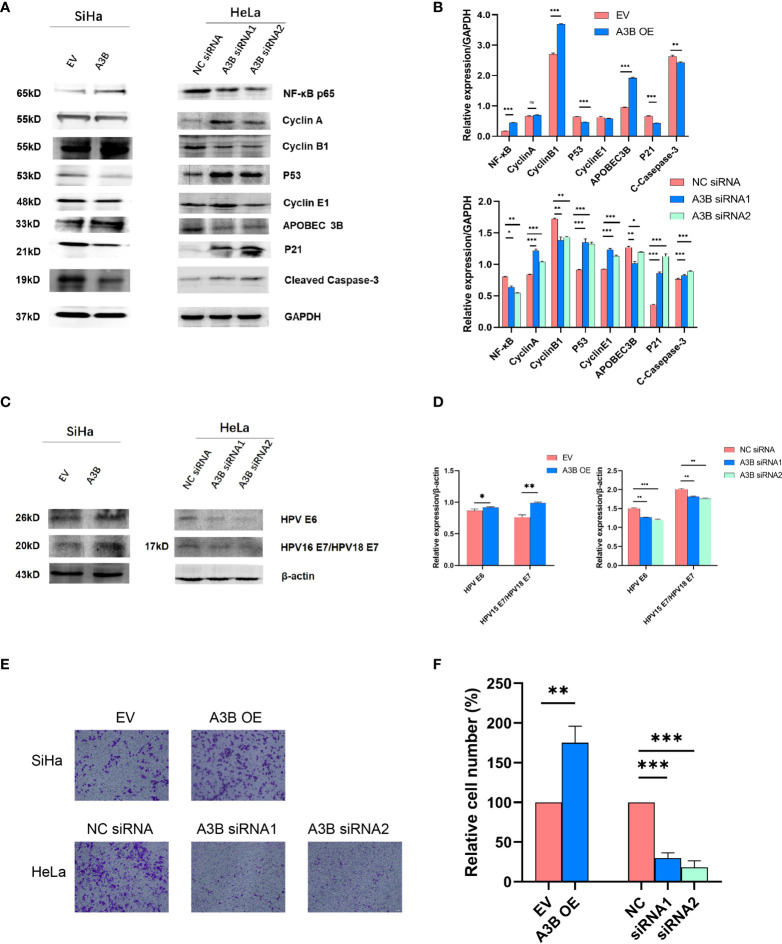
APOBEC3B is associated with cell motility and affects the p53 signal pathway. **(A, C)** Western blot assay results of p53 pathway-associated proteins, cell cycle proteins, E6, and E7 were affected by *A3B* expression levels. A3B reduced the protein levels of p53, cyclin E1, and p21 in both SiHa and HeLa cells. GAPDH was used as a loading control. **(B, D)** Quantification of Western blot assay data. **(E)** The migrating ability of HeLa and SiHa cells was associated with A3B expression levels, monitored by using a transwell migration assay. A representative image of migrated cells; 3.0 × 10^3^ cells were seeded into the upper chamber. Scale bar:50 μm. **(F)** Statistical analysis of **(E)**. ^*^ *p* < 0.05; ^**^ *p* < 0.01; ^***^ *p* < 0.001.

**Figure 5:**
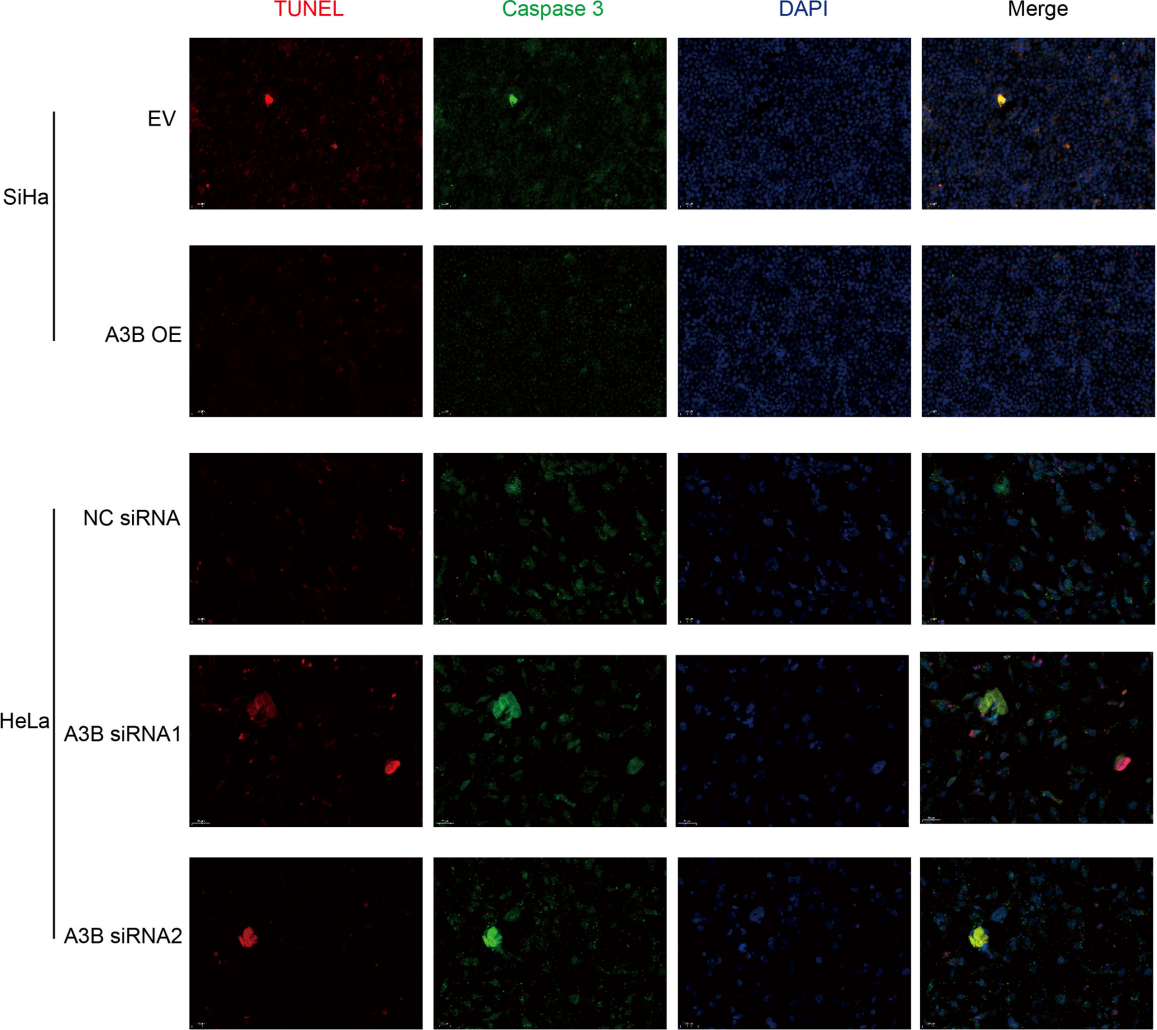
Relationship between apoptosis-associated proteins and protein expression of A3B. Representative image of fluorescence. Red represents TUNEL, green represents caspase-3, and blue represents DAPI. A3B correlates inversely with the prevalence of cervical cancer apoptosis.

**Figure 6:**
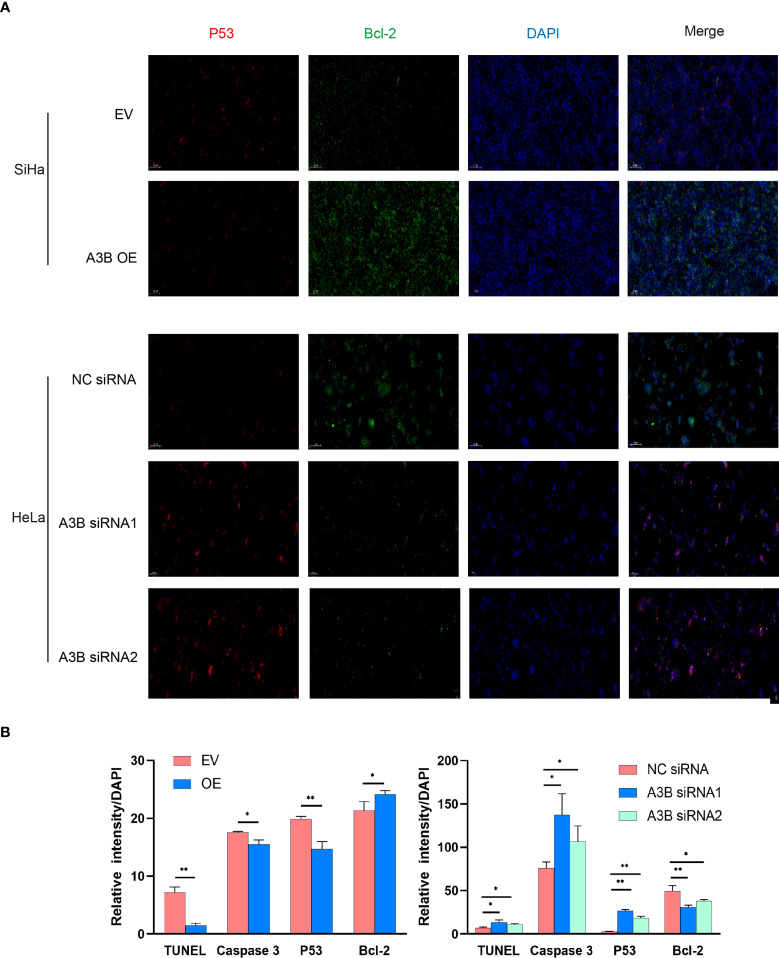
Relationship between p53, Bcl2, and protein expression of A3B. **(A)** Representative image of fluorescence. Red represents p53, green represents Bcl-2, and blue represents DAPI. A3B correlates inversely with the expression of p53 and positively with Bcl-2 in cervical cancer. **(B)** Quantification of immunofluorescence data. ^*^ *p* < 0.05; ^**^ *p* < 0.01.

To investigate the effect of APOBEC3B on cervical cancer progression, transwell migration and immunofluorescence staining were conducted. SiHa cells were transfected with a plasmid expressing APOBEC3B or an empty vector (EV). HeLa cells were transfected with two independent siRNAs targeting human APOBEC3B (siA3B-1 and siA3B-2). As shown in **Figure 4A, B**, the expression of APOBEC3B protein decreased significantly after transfection with siRNA but increased remarkably after transfection with a plasmid expressing APOBEC3B, which confirmed the effectiveness of our gene knockdown and overexpression method. As shown in **Figures 4E, F** APOBEC3B overexpression demonstrated a positive effect on the migratory capacity of SiHa cells compared with the empty vector, while APOBEC3B knockdown inhibited the migratory capacity of HeLa cells compared with the negative control (NC). Subsequently, immunofluorescence staining of apoptosis-associated proteins, including Bcl-2 and caspase-3, as well as TUNEL staining were performed to explore the effect of APOBEC3B on cell apoptosis. As shown in **Figure 5**, fewer TUNEL-positive cells were detected in the APOBEC3B overexpression group than in the EV group, while more TUNEL-positive cells were detected in the APOBEC3B knockdown group than in the NC group. Furthermore, fewer caspase-3 cells were positive in the APOBEC3B overexpression group than in the EV group, while more caspase-3-positive cells were detected in the APOBEC3B knockdown group than in the NC group. Similar results in immunofluorescence of Bcl-2 could also be observed in **Figure 6A**. Quantification of immunofluorescence staining is shown in **Figure 6B**. Cleaved caspase-3 was also investigated by Western blot (**Figure 4A**). A3B overexpression led to a significant reduction of cleaved caspase-3, which implied that A3B may act as a negative regulator of apoptosis. All these findings suggest that APOBEC3B was an important regulator of apoptosis in cervical cancer cells and was associated with the malignant phenotype of cervical cancer cells.

### Effects of APOBEC3B expression on the viability of cervical cancer cells

It is suggested that APOBEC3B may play an important role in cancer progression (23); therefore, we observed whether APOBEC3B promoted cell viability of human cervical cancer cells. As shown in **Figures 8A, B**, APOBEC3B overexpression led to elevated cell viability in SiHa cells compared to the control groups, and A3B knockdown contributed to a decrease in cell viability in HeLa cells compared to the negative control groups (**Figures 8C, D**). It is speculated that the expression of A3B might influence the cell cycle and the apoptosis of cervical cancer (15). Western blot was performed to detect the protein expression levels of p53-mediated signaling pathways p53, p21, cyclin A, cyclin B1, cyclin E1, and NF-kB, which were relevant to the proliferation of tumor cells (25–27) (**Figures 4C, D** **)**. As shown in **Figure 4A**, we found that silencing APOBEC3B would promote the expression of p53. At the same time, the expression of cyclin A, cyclin E1, and p21 was significantly upregulated, which were critical cell cycle proteins for cell proliferation (28–31). Considering the strong relationship of E6 and E7 proteins with A3B, we also tested the expression of E6 and E7 proteins (**Figures 4C, D** **)**. A3B overexpression led to upregulation of E6 and E7 proteins, while A3B knockdown showed opposite results. Therefore, A3B may promote p53 degradation by upregulating the E6 protein. The elevated p21 is consistent with increased p53 protein upon A3B knockdown in the HeLa cell line. Conversely, both p53 and p21 proteins are reduced in A3B over expressing SiHa cell line.

**Figure 7:**
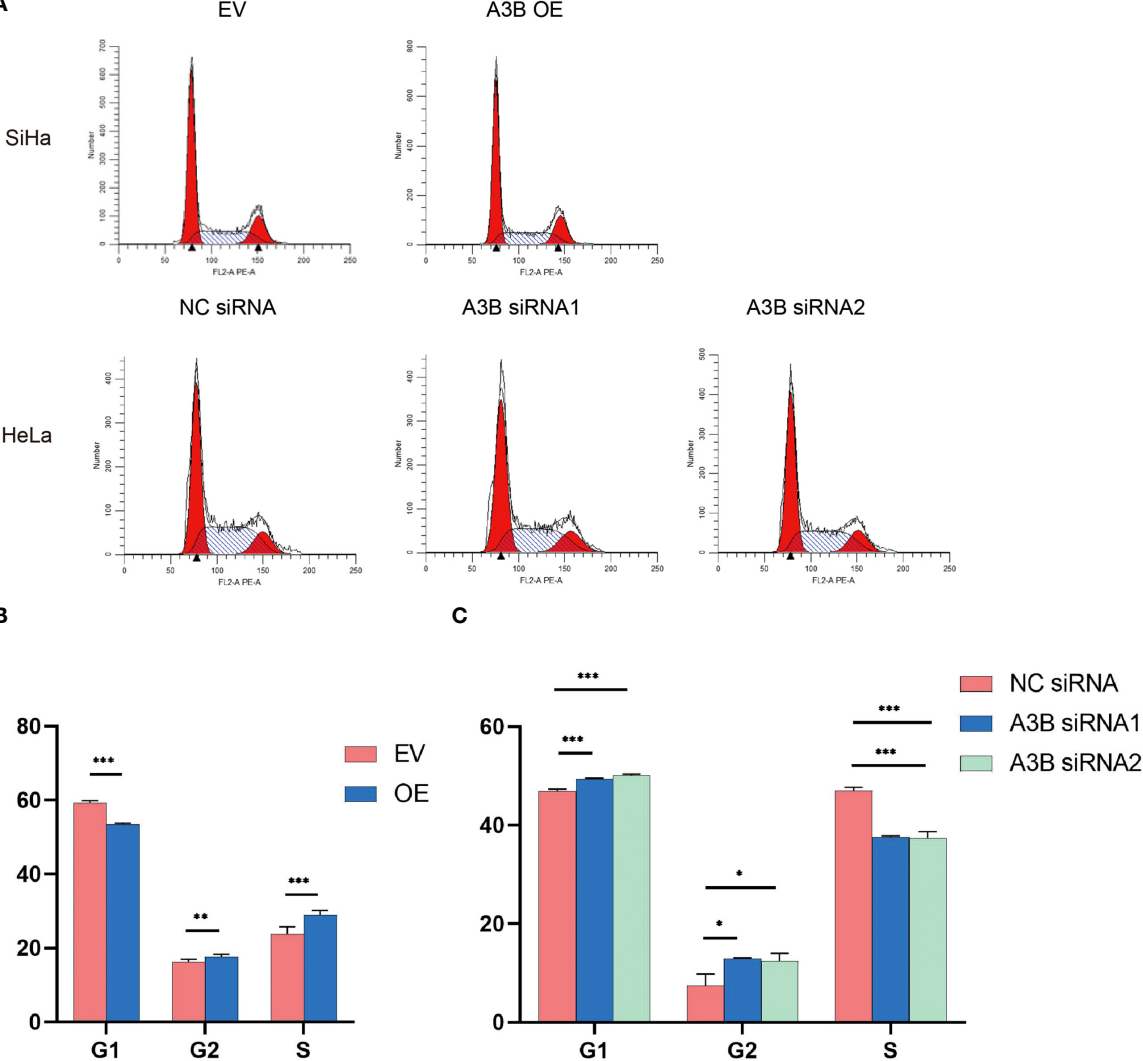
Effects of A3B on the number of cervical cancer cells in S- and G2/M-phases. **(A)** Typical flow cytometric analysis of the cell cycle. **(B)** Statistical analysis of SiHa cells. **(C)** Statistical analysis of HeLa cells. Apparently, A3B influences cervical cancer cells in the G1-, S-, and G2-phases. ^*^ *p* < 0.05, ^**^ *p* < 0.01, ^***^ *p* < 0.001.

The cell cycle protein-dependent kinase (CDK) inhibitor (p21) is an important negative regulator in cell cycle regulation. In response to DNA damage, it is stimulated by p53, which blocks the cell cycle (32). It is considered to be a tumor suppressor due to its ability to block cell cycle progression (33). Elevated levels of cell cycle proteins D1 and E have been observed in tumors, which can lead to uncontrolled cell proliferation. The active cell cycle protein/CDK complex can be regulated by binding to CDK inhibitors, thereby inhibiting cell cycle progression from G1- to S-phase (34). The result shows that overexpression of A3B in SiHa increased E7 protein and A3B siRNA reduced E7 protein in the HeLa cell line. It is known that E7 is responsible for G1-S transition by binding and degrading pRB pocket proteins. The active pRB normally downregulates the expression of cyclins and S-phase genes in the G1-phase of the cell cycle. Thus, the change in the protein level of cyclins and CDKs in response to A3B modulation may be due to altered E7 proteins. NfkB may have a supplementary role in regulating cyclins and CDKs (35, 36). However, reduced NfkB in the A3B knockdown HeLa cell line may have promoted the apoptotic pathway as indicated by elevated cleaved caspase-3. Flow cytometry (FCM) results demonstrated that upregulating A3B led to a significant S and G2 upregulation (**Figures 7A–C**), while A3B knockdown contributed to an increase in the G1-phase and a reduction of the G2-phase. Both results have indicated that APOBEC3B is related to the cell cycle thus promoting cell proliferation.

### APOBEC3B contributes to chemoresistance in cervical cancer cells

**Figure 8:**
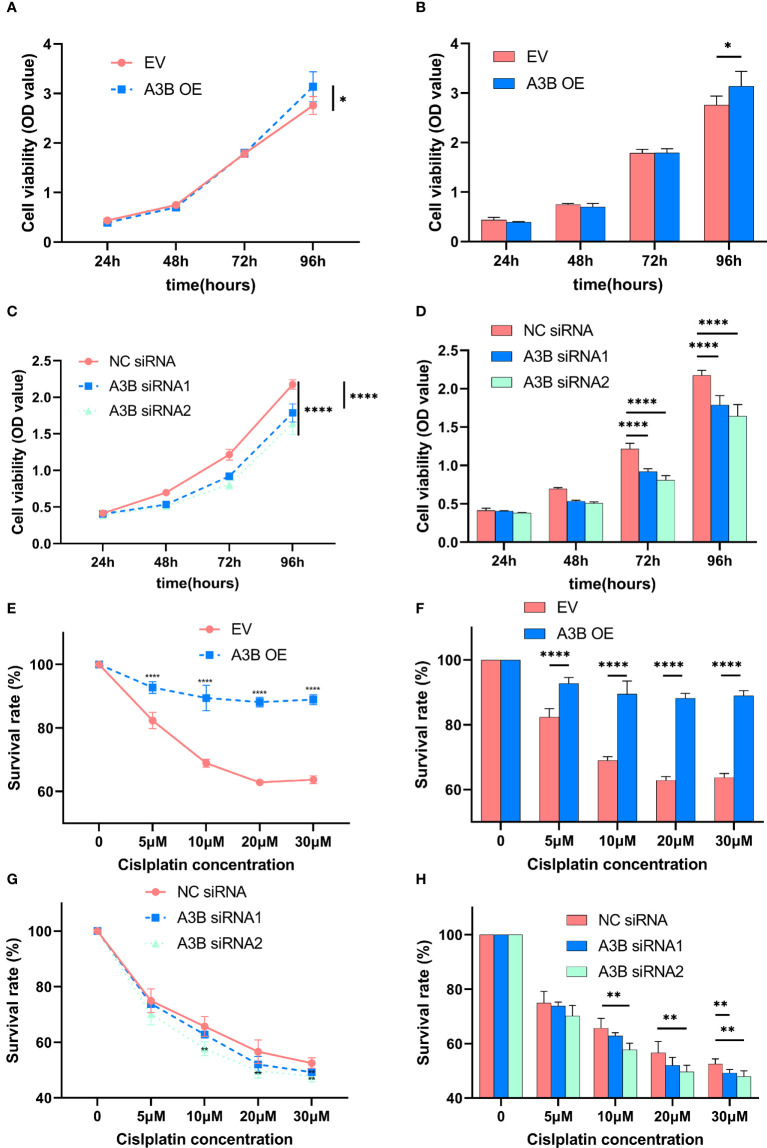
Effects of A3B on the proliferation and chemoresistance of cervical cancer. **(A, C)** A3B promotes SiHa and HeLa cell proliferation *in vitro*. **(B, D)** Statistical analysis of **(A, C)**. **(E, G)** A3B expression promotes cisplatin resistance in SiHa and HeLa cell *in vitro*. **(F, H)** Statistical analysis of **(E, G)**. ^*^ *p* < 0.05; ^**^ *p* < 0.01; ^****^ *p* < 0.0001.

In order to explore the effect of APOBEC3B on cytotoxicity drugs, we then treated SiHa cells and HeLa cells with different concentrations of cisplatin. Cell proliferation was determined by the CCK8 assay at 24, 48, and 72 h. As shown in **Figures 8E–H**, cervical cancer cell lines with high expression of A3B have lower sensitivity to cisplatin in a time- and concentration-dependent manner. Therefore, A3B may be a promising therapeutic target for cervical cancer.

## Discussion

Cervical cancer is still the most common gynecologic cancer among women and is a major cause of female cancer-related deaths worldwide. Cervical cancer remains an important health burden for developing countries (1). Accordingly, effective treatments are urgently needed. Recent research has illustrated that APOBEC3B, which has a unique role in the cancer cell cycle, might be a potential therapeutic target in the treatment of cancer (37–41).

It is well known that APOBEC3B plays a crucial role in retrovirus and endogenous retrotransposon restriction by hyperediting complementary DNA (cDNA) intermediates (42). Increasing research has shown that APOBEC3B may be a predominant mutagenic factor influencing the occurrence and evolution of various cancers (43) such as breast cancer (44), gastric cancer (45), chondrosarcoma (46), hepatocellular carcinoma (47), and so on. However, its potential role in cervical cancer is still not fully understood.

The aim of this study was to evaluate the potential value of A3B in cervical cancer by combining bioinformatic analysis with *in vitro* experiments. The bioinformatic results showed that A3B was highly expressed in malignant tumors. GEO analysis showed that HPV infection could not cause an increase in A3B expression levels. KEGG and GO analyses are displayed above. In addition, we scored tumor stemness, which is an evaluation of the proliferative activity and malignant potential of tumor cells, on samples with different levels of A3B expression. Both results suggested that A3B was likely to be an important factor in the development and progression of cervical carcinoma.

*In vitro*, we found that A3B could promote cell proliferation and migration and affect apoptosis. The results of immunofluorescence revealed alterations in TUNEL, caspase-3, and Bcl-2. Together with cleaved caspase-3, all the data revealed that A3B was a negative regulator of apoptosis. Immunoblot results showed that A3B induced NF-Kb, cyclin A, cyclin B1 and reduced cyclin E1. Furthermore, p53 was upregulated by A3B through E6. Flow cytometry results have indicated that A3B OE increased S-phase and reduced G1-phase, while its silencing prolonged G1 and G2 but reduced S-phase. Thus, A3B expression favors cell proliferation. Chemoresistance experiments showed that cervical cancer cell lines with high expression of A3B have lower sensitivity to cisplatin.

However, based on our clinical data, no overall survival difference was observed between the A3B^high^ group and the A3B^low^ group. The following reasons could be possible explanations. First, cervical cancer has a relatively good prognosis with a low mortality rate. Second, our clinical sample size is relatively small (*n* = 90), thus having no adequate power.

In conclusion, our data demonstrate that A3B was overexpressed in cervical cancer and promoted proliferation of cervical cancer cells through the regulation of HPVE6 and HPVE7 protein levels, as well as the regulation of cell cycle, p53 pathway, and apoptosis. We revealed the potential role of A3B in cervical cancer, and it may be a promising therapeutic target for cervical cancer.

## Data availability statement

The raw data supporting the conclusions of this article will be made available by the authors, without undue reservation.

## Ethics statement

Written informed consent was obtained from the individual(s) for the publication of any potentially identifiable images or data included in this article.

## Author contributions

ZW and JG have contributed equally to this work and share first authorship. All authors contributed to the article and approved the submitted version.

## Funding

This work was funded by the National Natural Science Foundation of China (Grant Number: 81973119) and the Shanghai Talent Development Fund (Grant Number: 2017090).

## Conflict of interest

The authors declare that the research was conducted in the absence of any commercial or financial relationships that could be construed as a potential conflict of interest.

## Publisher’s note

All claims expressed in this article are solely those of the authors and do not necessarily represent those of their affiliated organizations, or those of the publisher, the editors and the reviewers. Any product that may be evaluated in this article, or claim that may be made by its manufacturer, is not guaranteed or endorsed by the publisher.

## Supplementary material

Supplementary Figure 1
**(A)** The relationship between APOBEC3B and tumor stemness. **(B)** Venn diagram of 39 common upregulated genes and 1 common downregulated gene between TCGA and GSE26511 **(C)** Commonly upregulated genes Metascape analysis. **(D)** Expression level of A3B in different cell lines. **(E)** Cell viability assay of HeLa, Hela NC siRNA, HeLa A3B siRNA1, and HeLa A3B siRNA2. ^*^ *p* < 0.05, ^**^ *p* < 0.01, ^***^ *p* < 0.001, ^****^ *p* < 0.0001.
