# Supplementary figures and images for: APOBEC3B is overexpressed in cervical cancer and promotes the proliferation of cervical cancer cells through apoptosis, cell cycle, and p53 pathway
Source: Front Oncol. 2022 Sep 29;12:864889. doi: 10.3389/fonc.2022.864889 (PMC9556651; doi:10.3389/fonc.2022.864889)

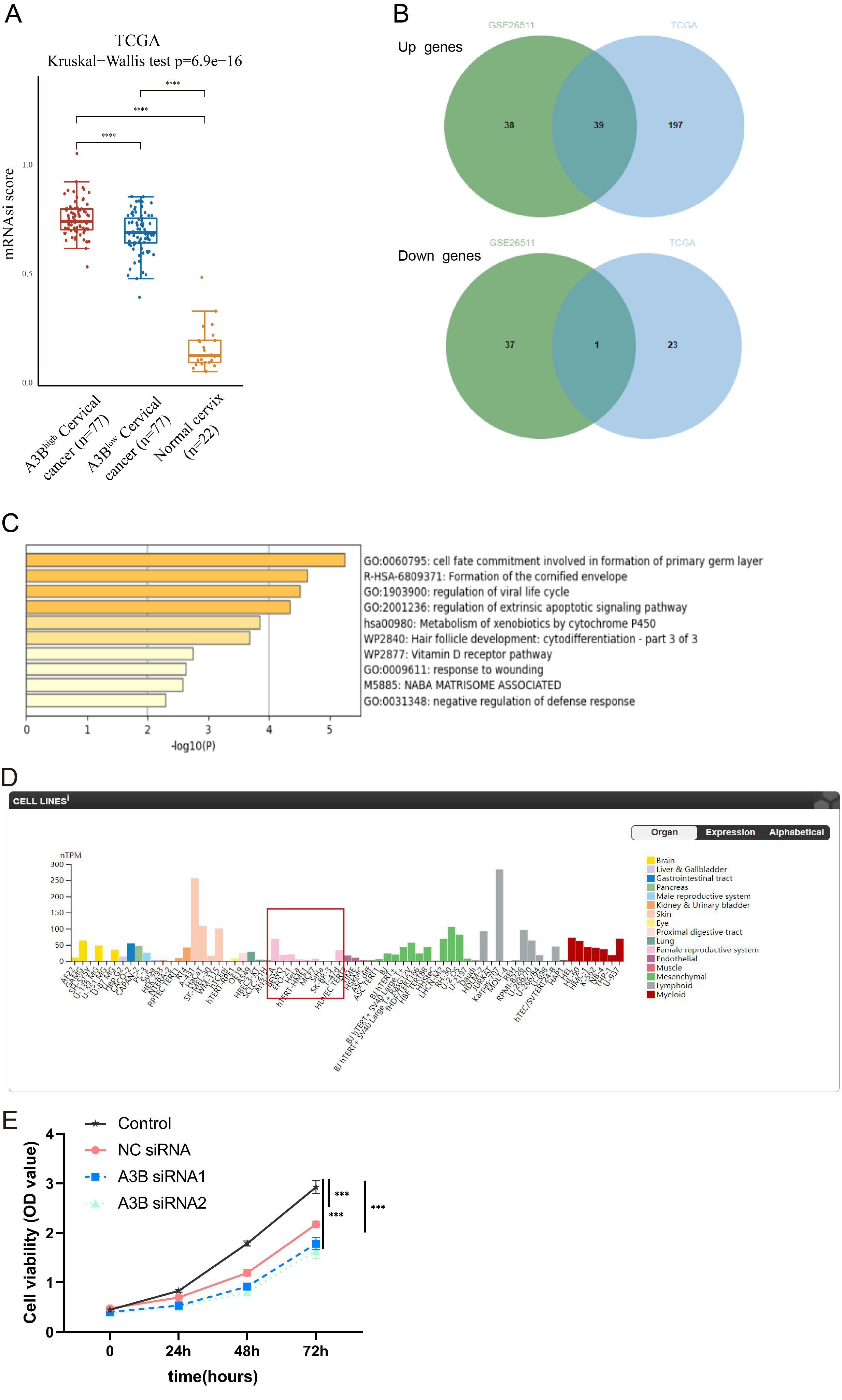

Supplement: Supplementary Figure 1 — (A) The relationship between APOBEC3B and tumor stemness. (B) Venn diagram of 39 common upregulated genes and 1 common downregulated gene between TCGA and GSE26511 (C) Commonly upregulated genes Metascape analysis. (D) Expression level of A3B in different cell lines. (E) Cell viability assay of HeLa, Hela NC siRNA, HeLa A3B siRNA1, and HeLa A3B siRNA2. * p < 0.05, ** p < 0.01, *** p < 0.001, **** p < 0.0001. [file Image_1.jpeg]
