# Supplementary material for: APOBEC3B is overexpressed in cervical cancer and promotes the proliferation of cervical cancer cells through apoptosis, cell cycle, and p53 pathway
Source: Front Oncol. 2022 Sep 29;12:864889. doi: 10.3389/fonc.2022.864889 (PMC9556651; doi:10.3389/fonc.2022.864889)
Supplement: Supplementary file 2 [file Table_1.docx]

TableS1.Common different expression genes of TCGA and GSE26511

| Up-regulated genes | Down-regulated gene |
| --- | --- |
| APOBEC3B | SPINK6 |
| ZFR2 |  |
| KRT15 |  |
| SYNGR3 |  |
| FOXN1 |  |
| GABRP |  |
| TRIM59 |  |
| DMRT2 |  |
| PAX9 |  |
| PRSS12 |  |
| SYCP2 |  |
| EGFL6 |  |
| PMAIP1 |  |
| SERPINB13 |  |
| KRT13 |  |
| CX3CL1 |  |
| SOX2 |  |
| APOBEC3A |  |
| SUSD4 |  |
| NDRG4 |  |
| CYP2S1 |  |
| PIR |  |
| CAPNS2 |  |
| ABCA13 |  |
| P2RY1 |  |
| GBP6 |  |
| ADH7 |  |
| TMPRSS4 |  |
| EYA2 |  |
| KBTBD12 |  |
| KLF4 |  |
| ALDH3A1 |  |
| DAPL1 |  |
| TNFSF10 |  |
| FAT2 |  |
| AKR1B10 |  |
| DSC3 |  |
| KRT5 |  |
| CYP4X1 |  |
